# Dynamically generating T32 training documents using structured data

**DOI:** 10.5195/jmla.2019.401

**Published:** 2019-07-01

**Authors:** Paul James Albert, Ayesha Joshi

**Affiliations:** Samuel J. Wood Library, Weill Cornell Medicine, New York, NY, paa2013@med.cornell.edu; Weill Cornell Graduate School, Weill Cornell Medicine, New York, NY, ayj2002@med.cornell.edu

## Abstract

**Background:**

The US National Institutes of Health (NIH) funds academic institutions for training doctoral (PhD) students and postdoctoral fellows. These training grants, known as T32 grants, require schools to create, in a particular format, seven or eight Word documents describing the program and its participants. Weill Cornell Medicine aimed to use structured name and citation data to dynamically generate tables, thus saving administrators time.

**Case Presentation:**

The author’s team collected identity and publication metadata from existing systems of record, including our student information system and previous T32 submissions. These data were fed into our ReCiter author disambiguation engine. Well-structured bibliographic metadata, including the rank of the target author, were output and stored in a MySQL database. We then ran a database query that output a Word extensible markup (XML) document according to NIH’s specifications. We generated the T32 training document using a query that ties faculty listed on a grant submission with publications that they and their mentees authored, bolding author names as required. Because our source data are well-structured and well-defined, the only parameter needed in the query is a single identifier for the grant itself. The open source code for producing this document is at http://dx.doi.org/10.5281/zenodo.2593545.

**Conclusions:**

Manually writing a table for T32 grant submissions is a substantial administrative burden; some documents generated in this manner exceed 150 pages. Provided they have a source for structured identity and publication data, administrators can use the T32 Table Generator to readily output a table.

## BACKGROUND

The US National Institutes of Health (NIH) provides funding to academic institutions for training doctoral (PhD) students and postdoctoral fellows. These grants are called “training grants” or T32 grants. T32 grants are prestigious awards for academic institutions that reflect the quality of training, quality of mentoring, and success of graduate programs [1]. According to NIH RePORTER, as of September 2017, 256 different institutions were recipients of the 2,630 active T32 training grants [2].

To evaluate an existing or consider a new training program, NIH requires potential awardees to report on their programs’ outcomes in the form of seven or eight data tables, depending what type of trainee and whether the application is a new one or a renewal [3]. Elements include trainee characteristics, trainee publications, mentoring records, and funding of faculty mentors. Each table requires supplying certain data in a particular format.

Typically, the submission process begins in earnest when a program leader designates a list of faculty to serve as mentors for a training grant. Then, a program administrator coordinates collection of information about all the trainees of a mentor in the past ten years, including mentees from prior affiliations in some cases. For all trainees, administrators need to know the date range of their mentor-mentee relationships as well as any publications that they have authored [3].

**Figure 1:**
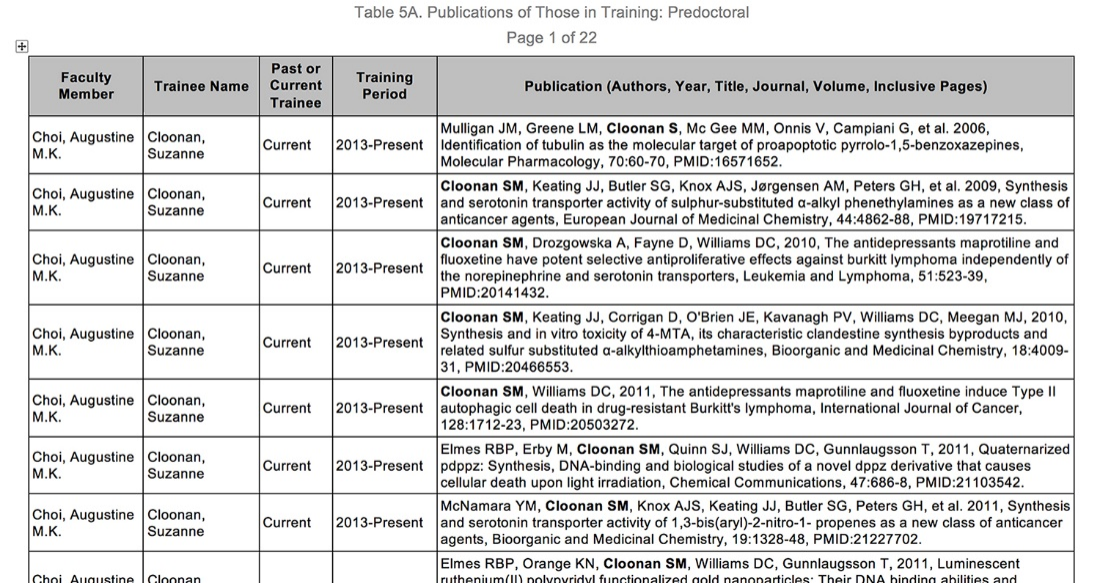
Sample output of Table 5A for T32 reporting

Collecting information and manually generating these tables represents a sizable administrative burden. Before a submission, information has to be requested from investigators, institutional grant administrators, institutional reporting, and other sources. Collected information has to be collated and manually entered into 7 or 8 Microsoft Word documents, and some of the tables, especially Table 5, potentially exceed 150 pages (Figure 1). Some faculty are listed on multiple T32 grants, others need to be removed or added at the last minute, and the names of all mentees need to appear in bold. In Table 5, administrators must list participating faculty, their mentees (including those from prior affiliations, for whom data are sparse), their training periods, and authored publications [3].

According to NIH documentation, “[the] Public reporting burden for this collection of information is estimated to average 4 hours per response [grant submission]” [3]. In the experience of the author’s institution, producing a new or renewal submission for T32 funding can take around three months, involve coordinating feedback from more than a dozen people, and require the exchange of sixty or more email messages.

Other institutions have complained about the volume of work required to produce a T32 submission. According to one program administrator at a recipient institution:

I think most people will tell you that putting together the application for a training grant renewal is probably the least pleasant experience you’ll ever have. People would rather have a root canal without anesthesia than put one of these grants together, because the documentation and requirements of what you have to present are just enormous. [4]

Over the course of at least the last ten years, the Samuel J. Wood Library at Weill Cornell Medicine has fulfilled a variety of publication reporting requests for various departments. In October 2016, a member of the Graduate School administrative staff asked colleagues where she could get help with publication reporting, and several recommended the library. After an initial discussion, the library began a collaboration with administrators in the Graduate School and the medical doctor (MD)-PhD and postdoctoral training programs.

The team’s proximate goal was to save administrators time by using structured identity and publication data to generate Table 5 dynamically, both for the upcoming grant submission cycle and for all subsequent ones. Given how many institutions like Weill Cornell Medicine already maintain structured identity and publication metadata for investigators and, to a lesser extent, for trainees [5], these data could be used for producing lists of publications in NIH’s specified Table 5 format.

## CASE PRESENTATION

We began by collecting data from our institution’s five prior T32 submissions. We copied content from our legacy tables into two temporary spreadsheets: one for people subdivided by faculty and mentees and the other for publications.

As mentors and mentees might be listed on multiple grants, we attempted to assign existing institutional person identifiers. This step revealed dramatic shortcomings in the availability and quality of Weill Cornell Medicine’s identity data. Only 39.2% of mentees had a unique institutional ID in Weill Cornell’s back-end student information system, 23.8% had a unique ID but only in the enterprise directory, and 36.9% did not have an institutional ID of any kind. The latter group largely included students who trained at a faculty’s previous institution. One faculty mentor was assigned 4 distinct institutional identifiers: the first in 2004, 2 in 2010, and another in 2012. For all prior publications, we systematically identified unique identifiers, such as PubMed identifiers (PMIDs) and digital object identifiers (DOIs).

Drawing on data from our enterprise directory, student information system, and a spreadsheet maintained on the shared drive of the Office of Postdoctoral Affairs, we updated the list of mentors, mentees, the date range of their mentorships, and mentees’ current status. Previous T32 submissions contained last name and first initial, so in cases in which we had no identity record, we needed to infer a mentee’s full name by looking at their known publications. Altogether, this manual clean-up process took approximately forty hours.

For identification of additional publications, we used ReCiter [6], an open source author disambiguation engine. ReCiter works by using institutional identity information (e.g., email address, name of potential coauthors such as the name of a mentor) to suggest candidate publications. Combining ReCiter with manual look-up in cases of common names, we were able to increase the number of known publications authored by students while they were active in the MD-PhD program from 724 to 1,095 and more still for students’ publications that they authored prior to their arrival at or after leaving our institution.

**Figure 2:**
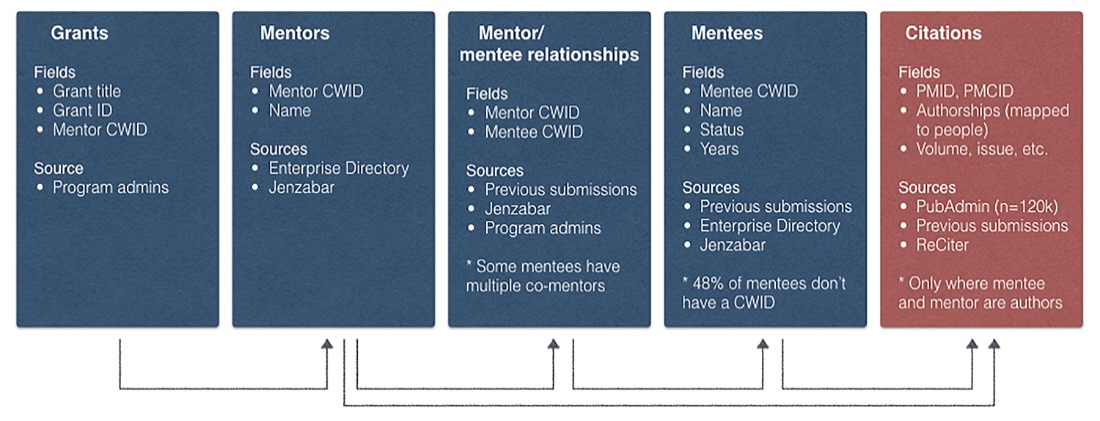
Data flow for generating T32 grant documents The database tables for generating T32 documents can work in any MySQL installation. The student identity data listed here comes from Jenzabar, a commercial software package adopted by Weill Cornell Medicine.

All identity and publication metadata were stored in a MySQL database. For this proof-of-concept, our data model was relatively simple, containing five tables (Figure 2):

- T32demo_grants: grants and mentors listed on the grants
- T32demo_mentors: identity information for mentors
- T32demo_mentors_mentees: mentor/mentee relationships
- T32demo_mentees: identity information for mentees
- T32demo_citation: citation information, a unique record is required for each author, and the target author is delimited by braces (e.g., Ryter SW, Koo JK, {Choi AMK})

After assembling the relevant identity data, we worked with program administrators and directors to confirm the list of mentors and mentees who were in scope.

Upon creation of the document, we shared it with program representatives and invited their feedback on data quality. No program representative had access to the database, but we insisted that any data errors that they noticed needed to be updated in the source system. Otherwise, we would lose the benefit of this feedback.

It is considered best practice to use a programming language like Java to output XML [7]. However, as we needed to cater to available expertise and merely provide a proof-of-concept, we wrote a query using the MySQL query language. With this query, we only needed to provide an identifier for the grant. The query retrieves the faculty mentors listed on the grant, their mentees, and the publications authored by those mentees. The information is output into a Word XML document, which can be downloaded and opened in Word. Various limits, such as whether both mentor and mentee need to be listed as coauthors, can be invoked in the query itself. Transitioning this code from MySQL to Java should be straightforward for any experienced developer.

We generated the T32 training document using a query that ties faculty listed on a grant submission with publications that they and their mentees authored, bolding author names as required. Because our source data are well-structured and well-defined, the only parameter we needed to provide in the query is a single identifier for the grant itself. The open source code for producing this document is at http://dx.doi.org/10.5281/zenodo.2593545 [8].

**Figure 3:**
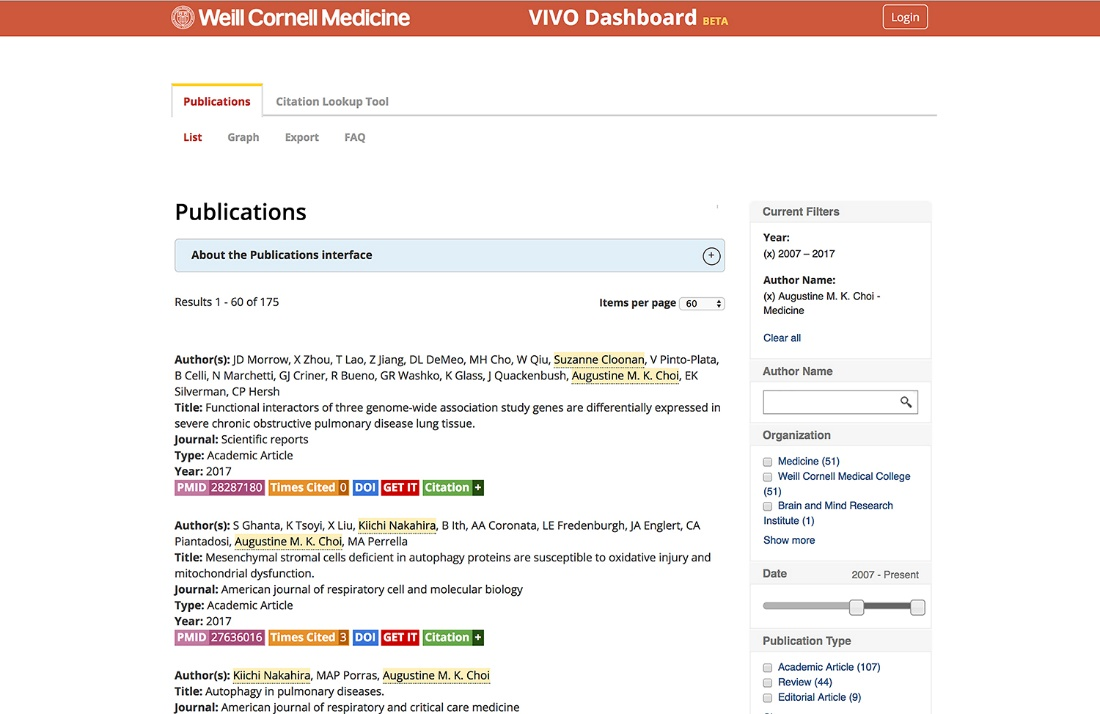
VIVO Dashboard, a tool for reporting on publications

We successfully completed our intended goal of dynamically generating Table 5 for T32 grant submissions. Thus far, this method has been used for four new submissions, three of which were resubmitted and two of which were funded. We are now able to add and remove mentors from a T32 grant submission, with changes reflected when the Word file is regenerated. Because the publication data came from a centrally maintained source system, the feedback we received during this process will be used to update other systems: VIVO, our public-facing researcher profile system; the Faculty Review Tool, a homegrown application for evaluating faculty contributions in a given year; and VIVO Dashboard, our publication reporting tool (Figure 3) [9]. Further, this work gave us an impetus to resolve dozens of cases in which an individual was missing an institutional identifier or had been assigned multiple identifiers.

## DISCUSSION

This effort to dynamically generate Table 5 for T32 submissions exposed a weakness in the way our institution tracks mentorship. The institution maintains a central system for tracking mentorship relationships, but only for graduate and MD-PhD students who have been active since 2012. Other mentor/mentee relationships, such as those for postdoctoral fellows and MD-PhD students, are not tracked in any central system. It is also problematic that we have yet to offer program administrators and faculty mentors a user interface for viewing and providing feedback on their mentor-mentee relationships and publications. This step alone might have halved the messages sent between faculty mentors and program administrators. Some schools such as the University of California at San Francisco, in the form of its “3TS” application that sits on top of SalesForce, have built such interfaces.

To meet these challenges, our institution is striving to ensure that every individual has a unique identifier, to capture relevant data in centrally maintained systems defining where certain data are authoritative, to provide a user interface for authorized users to view and correct these data, and to share these data so that they can be reused in other systems. This final step, in particular, promises to reduce administrative burden, while also improving data quality. As we discovered in other circumstances, the best way to improve the quality of data in a system is to use it. Unfortunately, it seems possible that a large number of T32 awardees do not reuse the data to produce Table 5 for other uses, such as profile systems. If this is the case, there is widespread administrative inefficiency among T32 recipients.
